# Ensuring children's inclusion in the Bundibugyo Ebola response: Striving to do better this time

**DOI:** 10.1371/journal.pgph.0007117

**Published:** 2026-09-02

**Authors:** Mike Kalmus Eliasz, Michael Adeyemi-Lawal, Lauren D’Mello Guyett, Daniela Manno, Bruce Myango, Paula Reges, Neal Russell

**Affiliations:** 1 London School of Hygiene and Tropical Medicine, London, United Kingdom; 2 The Pandemic Institute, University of Liverpool, Liverpool, United Kingdom; 3 Medecins Sans Frontieres Operational Centre Amsterdam, Amsterdam, The Netherlands; 4 Medecins Sans Frontieres, Kinshasa, Democratic Republic of Congo; 5 Instituto Nacional de Infectologia Evandro Chagas, Fundação Oswaldo Cruz (INI-Fiocruz), Rio de Janeiro, Brazil; Viborg Regional Hospital: Regionshospitalet Viborg, DENMARK

As of the 23rd August 2026 there are 5606 confirmed cases and 2683 confirmed deaths according to the World Health Organisation (WHO) in the current Bundibugyo outbreaks in the Democratic Republic of Congo, France and Uganda [1]. Of confirmed cases up to 5th August 2026 where complete age and sex information is available (3454 cases and 1124 deaths) children aged 0–4 years make up 10.0% of cases, yet 19% of deaths, with an estimated case fatality rate (CFR) of 61.7% as opposed to 28.8% for those aged 18 and over [2]. This may reflect differential reporting, though it is important to note that significantly higher CFR’s among children, particularly infants, have been observed in other Ebola outbreaks [3].

Experience from previous epidemics demonstrates how children are not only disproportionately affected by the indirect impacts of public health emergencies but are also late to benefit from access to medical countermeasures such as vaccines and therapeutics. Given ethical concerns surrounding inclusion of children in clinical research, especially regarding consent and their differing pharmacokinetics and immunological functions, as well as the urgency of research in an emergency, children, are frequently excluded from early trials of medical countermeasures. This is alongside pregnant women, breastfeeding mothers, and others deemed clinically vulnerable. Instead, after proving safety in healthy adults, the use of medical countermeasures is either usually progressively expanded outwards or there are delays in developing countermeasures specific for these groups. However, if they are at greater risk of negative outcomes from disease this does raise questions of is it ethical to delay their inclusion in studies

For therapeutics, children were included from the start in some clinical trials evaluating treatments for Ebola Virus Disease (EVD) caused by Zaire ebolavirus, as demonstrated by the PALM trial [4]. Two monoclonal antibody treatments (mAb114 and REGN-EB3) were evaluated in this trial and, based on the evidence generated, received a strong recommendation from WHO in 2022 for use in adults and children with confirmed EVD, as well as in neonates aged seven days or younger with unconfirmed infection who were born to mothers with confirmed EVD [5].

However, when it came to vaccines there have been significant delays in the inclusion of children in trials. The rVSV based Ervebo vaccine, tested during the was Ebola Ca-Suffit trial in 2015–16, initially excluded children, but inclusion was later revised down to age 6 years, after 4 months of enrolment following a data safety monitoring board review [6]. Younger children over 1, were included in Ervebo vaccination campaigns in the subsequent outbreak in DRC in 2018–2020, it is currently only licenced for children aged over 1 for the Zaire ebolavirus. A year into the outbreak SAGE guidelines were revised to allow the inclusion of infants aged 6–12 months, pregnant and breastfeeding women [7]. Phase 2 trials were undertaken in infants aged 4 months and above of a different vaccine in West Africa, after the epidemic had ended, of the Ad26.Zebov and the MVA-BN-Filo vaccines showing safety and immune response which whilst encouraging was not available during the emergency [8].

Other Public Health Emergencies of International Concern (PHEICs) have demonstrated how late the inclusion of children can be, even when vaccines with prior usage in children exist. Despite the declaration of PHEICs in 2022 and 2024 for Mpox it was only in 2025 that safety and efficacy studies for use in pregnancy and infancy were planned for the MVA-BN vaccine [9]. Additional delays in emergency approvals for off-label use in country and availability of the alternative vaccine with an existing licence for children (LC-16), meant that children were not included in vaccination response in the 2024 outbreak until almost 200 days into the outbreak, despite children making up the majority of deaths [9].

On 28^th^ May, 2026, the WHO R&D blueprint produced a list of the most likely candidates for treatment of and prevention of Bundibugyo virus disease, however it did not explicitly mention in the meeting summary use of these tools in children (10). WHO are currently recommending three potential candidate vaccines including the Oxford/Serum Institutes ChAdOx1, IAVI’s rVSV vaccine and Ervebo (as evidence of some cross protection from animal studies). Doses are now available for the Oxford/Serum Institute Vaccine which has entered phase 1 trials, however the IAVI vaccine was estimated to take 7–9 months to produce [10,11]. In addition, Moderna received funding from CEPI for a mRNA vaccine in June 2026 [11]. All three vaccine technologies have evidence of use in children for other indications.

For therapeutics, three therapeutics have been recommended including the monoclonals MB134 and maftivimab, either individually or in combination with the antiviral remdesivir. It has been confirmed that children of all ages are being enrolled in the Partners trial of these therapeutics for confirmed disease [10,12]. The antiviral obeldisivir was recommended by WHO for study, and the EBO-PEP study has commenced, as post exposure prophylaxis in tablet form but only for children over 12 [11]. Although, there is evidence to date that paediatric dosing of Obeldesivir has been used experimentally in younger children to treat for respiratory syncytial virus (RSV) and COVID-19 [10,13]. As part of the study children under 12 and pregnant women will be offered, on compassionate use, remdesivir, which is only available in intravenous form with the associated challenges and risks of a course of intravenous therapy and inpatient treatment in an outbreak setting [11].

Given the scale of the outbreak, is it possible to do better for children with regards to early access to the full range of post exposure prophylaxis tools and vaccines for Bundibugyo virus disease. Are there opportunities to undertake parallel studies, prepare paediatric formulations, identify dosing for younger children and undertake pharmacokinetic modelling and immunological response studies now before this outbreak grows?

It is important not to forget the context in which this outbreak is unfolding. The DRC is already home to some of the most acutely vulnerable children, faces not only one of the highest under-5 mortality rates globally, but simultaneous crises of measles, cholera, acute malnutrition and armed conflict [14]. Children in Ituri and surrounding provinces carry this burden before a single case of Ebola is counted. The question before the international community is not only whether children can be safely included in countermeasure trials, but also how to ensure their timely and equitable access to evidence based interventions and essential health services. Previous outbreaks have taught us that delays cost lives, and considerations are needed to simultaneously support access to other lifesaving services such as routine vaccination, nutrition, malaria prevention, education and protection services, noting recent evidence surrounding sexual violence against women and girls during outbreaks and conflict [15].

A PHEIC is also an emergency for children’s rights and survival. It is for decision makers in governments, organisations and UN agencies responding to the crisis to ensure that in protecting global health security, we protect children. As part of that a better treatment response for children is a moral imperative too.
